# Von Hippel-Lindau disease: pathophysiological and clinical advances

**DOI:** 10.1007/s10689-026-00579-8

**Published:** 2026-06-18

**Authors:** S. J. van Alfen, K. van der Tuin, B. van de Kooij, T. P. Links, W. T. Zandee

**Affiliations:** 1 Division of Endocrinology, Department of Internal Medicine, University Medical Center Groningen, University of Groningen, P.O. Box 30.001, 9700 RB Groningen, The Netherlands; 2 Department of Genetics, University Medical Center Groningen, University of Groningen, Groningen, The Netherlands; 3 Department of Medical Oncology, University Medical Center Groningen, University of Groningen, Groningen, The Netherlands

**Keywords:** Von Hippel-Lindau, Belzutifan, Hypoxia inducible factor, Hereditary cancer, Familial cancer syndrome

## Abstract

Von Hippel-Lindau (VHL) disease is a hereditary tumor predisposition syndrome caused by pathogenic germline variants in the *VHL* gene. Patients with VHL disease have an increased risk of developing characteristic VHL disease-associated lesions such as clear cell renal cell carcinoma, retinal angioma, central nervous system hemangioblastoma, pancreatic neuroendocrine tumors and pheochromocytomas. Loss of the *VHL* gene results in increased levels of the α-subunits of the heterodimeric hypoxia inducible factor (HIF). This drives transcription of HIF target genes which are involved in, amongst others: angiogenesis, erythropoiesis and iron metabolism. HIFs have been recognized as major drivers of VHL disease pathology and based on this notion, the HIF-2α inhibitor belzutifan was developed, which has marked a major breakthrough in the treatment of this disease. Belzutifan has now been approved for the treatment of a variety of VHL disease-associated lesions by the Food and Drug Administration and the European Medicines Agency. Interestingly, recent studies suggest that pVHL has functions beyond controlling HIF levels, and loss of these HIF-independent functions may further contribute to tumorigenesis in VHL disease. This review summarizes the most recent advances in pathophysiology, genotype–phenotype correlation, treatment guidelines, and potential future treatment options related to VHL disease.

## Introduction

Von Hippel-Lindau (VHL) disease is an autosomal dominant inherited tumor predisposition syndrome with an estimated prevalence of 1–39.000 to 1–91.000 [1]. Patients with VHL disease are prone to the development of one or multiple VHL disease-associated lesions, such as clear cell renal cell carcinoma (ccRCC), retinal angioma (RA), central nervous system (CNS) hemangioblastoma, pancreatic neuroendocrine tumors (pNET) and pheochromocytoma (PHEO). The disease is named after Eugen von Hippel (1867–1939), who described a patient with retinal angiomas in 1904, and Arvid Lindau (1892–1958) who was the first to describe a patient with multiple hemangioblastomas in the CNS in 1927. This eventually led to the development of the first clinical guidelines for VHL disease in 1964, but it was not before 1993 that the *VHL* gene was discovered [2, 3]. A clinical diagnosis of VHL disease can be made in either of the following cases: 1. A positive family history with at least one VHL disease-associated tumor, 2. At least two VHL disease-associated tumors, 3. At least two hemangioblastomas, 4. At least one hemangioblastoma and one other VHL disease-associated lesion. The exact criteria differ slightly amongst guidelines [4]. It is hypothesized that, in general, VHL disease-associated lesions develop after loss of the remaining wild-type *VHL* gene, following the “two-hit hypothesis” to inactivate tumor suppressor genes [5, 6]. This is supported with evidence for VHL disease-associated ccRCC, but initiating events are not fully understood in other VHL disease-associated pathologies.

Patients with VHL disease are often subdivided into two broad categories based on phenotype, mainly reflecting the risk of PHEOs. This concept arose in the late twentieth century when the framework was used by, amongst others, Chen et al. and Maher et. al. [7, 8]. Risk stratification is based on the specific *VHL* variant, combined with evidence from literature on the risk of these pathogenic variants for particular VHL disease-associated lesions. Those variants with a low risk for PHEOs are regarded to have type 1 VHL disease, whereas those with a high risk for PHEOs are seen as type 2. Type 2 is further subdivided based on either a low (type 2b) or high (type 2a) risk of ccRCC. Lastly, variants with very low risks for ccRCC or hemangioblastomas (HBs) and which are practically only associated with PHEOs are categorised as type 2c [4].

## Pathophysiology

### VHL function

The *VHL* gene encodes pVHL*,* which is foremost known for its role in promoting degradation of the α-subunits of the heterodimeric hypoxia inducible factor (HIF). pVHL interacts with the proteins elongin B and elongin C (ELOC) to form the VCB complex. Upon association with Cullin-2 and RBX1, the VCB complex functions as a multi-subunit ubiquitin ligase that targets the three HIFα family members (HIF-1α, HIF-2α and HIF-3α) for degradation. HIFα binds directly to pVHL, but this interaction requires HIFα to be hydroxylated by prolyl hydroxylases (PHDs). The activity of PHDs is dependent on oxygen, iron and 2-oxoglutarate. Under normoxic conditions, PHDs hydroxylate HIFα, targeting it for pVHL-mediated degradation. If oxygen levels are low (i.e*.* hypoxia), PHDs are not able to hydroxylate HIFα. Subsequently, HIFα accumulates, binds its partner HIF-1β (encoded by *ARNT*) and activates the transcription of over 5000 HIF-target genes (see also below) [9–13].

### Pathogenic VHL variants

The majority of pathogenic *VHL* variants result in HIFα stabilisation regardless of oxygen levels, thus causing a state known as pseudohypoxia. HIFs have several oncogenic properties that underly the increased risk for tumor formation as observed in VHL disease [9–11, 13]. Firstly, HIFs activate genes associated with the formation of new blood vessels such as vascular endothelial growth factor (*VEGF*). Hence, it is not surprising that many VHL disease-associated lesions, such as hemangioblastomas, are often highly vascularised [14]. Secondly, transcriptional activity of HIF-1α causes a shift from oxidative phosphorylation to glycolysis [13]. This metabolic rewiring is also known as the Warburg effect and has been observed in many tumors [15]. Several hypotheses have been put forward as to the exact benefits of the Warburg effect for tumor cell growth and survival. Glycolysis can be a fast ATP-producing energy source which does not require oxygen and produces carbon molecules which can be used for the synthesis of new lipids and proteins. In addition, the increased lactate production generates an acidic microenvironment, which dampens the anti-tumor immune response [16]. The theory of the Warburg effect in VHL disease-associated lesions is further supported by the observation that VHL disease-associated lesions have increased glucose transporter 1 (GLUT1) surface expression, which is consistent with high glucose consumption [17].

Other oncogenic functions of HIF have been described, although their relevance in VHL disease remains less well defined. For example, HIF overexpression has been correlated with increased metastatic potential in various malignancies [18]. Whereas this might contribute to disease severity in some cases, VHL disease-associated lesions are typically characterized by a low metastatic risk (e.g. in ccRCC, metastasis is rarely seen in lesions smaller than 3 cm) [19]. Furthermore, HIFs are involved in the expression of proliferation-promoting genes and in modulation of the immune system [20]. The latter is not only due to glycolysis-associated acidification, as described above, but HIF activity also directly dampens innate and adaptive immunity at the tumor site by driving transcription of immunosuppressive chemokines and of the inhibitory receptor programmed death ligand 1 (PD-L1) [21].

Interestingly, the oncogenic potential of either HIF-1α or HIF-2α varies per tumor location and stage, and either transcription factor can paradoxically also have tumor suppressor functions [22–30]. For example, HIF-2α is generally considered the oncogenic driver in ccRCC, whereas HIF-1α is considered to be dispensable, or even act as a tumor suppressor [25]. In agreement with the latter, about 30% of *VHL*-mutant ccRCC was found HIF-1α negative, and a recent genetic screen identified HIF-1α as the main suppressor of viability in *VHL*-mutated kidney cells [31, 32]. Nevertheless, in one recent mouse study, ccRCC initiation was driven by HIF-1α, rather than HIF-2α [25]. HIF-1α has also been proposed to function as a tumor suppressor in neuroblastomas, whereas HIF-2α may exhibit tumor-suppressive effects in non-small cell lung cancer and gliomas [22–30, 33].

### pVHL functions beyond HIF regulation

VHL disease is primarily associated with dysfunction of the VHL-HIF pathway. However, not all pathogenic *VHL* variants lose the ability to ubiquitinate HIFα. Most notably, type 2C associated pathogenic *VHL* variants can still completely suppress HIFα [12, 34, 35]. Hence, HIF-independent functions appear to drive VHL disease-related tumor formation in type 2C patients, and these functions might also contribute to tumorigenesis in other patients. Moreover, inhibition of HIF-2α using belzutifan effectively reduces tumor load in a large proportion of VHL disease patients, but not all patients respond [36]. This suggests that VHL disease-associated lesions can develop and grow in a HIF-independent manner. Delineating the HIF-independent drivers of pathogenic *VHL* variants tumor formation is essential for the development of therapeutic strategies to treat belzutifan non-responders.

Early studies on pVHL function identified an interaction between pVHL and fibronectin, an essential component of the extracellular matrix [37, 38]. Renal cell carcinoma cell lines and mouse embryonic fibroblasts that were *VHL* deficient had severely reduced fibronectin matrix assembly. Similarly, VHL disease-associated mutants of pVHL, including type 2C variants, lost the interaction with fibronectin [35]. The interaction with fibronectin is dependent on the post-translational modification of pVHL with the ubiquitin-like molecule NEDD8 [39]. Notably, a pVHL mutant that could not be NEDDylated failed to suppress tumor growth in an RCC cell line xenograft experiment, despite it being capable of HIF ubiquitination [39]. Together, these data suggest that pathogenic *VHL* variants could promote tumor formation in a HIF-independent manner by disrupting extracellular matrix formation.

More recently, a potentially targetable HIF-independent function of pVHL was indicated by a study showing that pVHL supports mitochondrial biogenesis [40]. The protein mitochondrial transcription factor A (TFAM), an essential activator of mitochondrial replication, was found to be hydroxylated by PHD3, driving a direct interaction with pVHL. Instead of targeting TFAM for ubiquitin-mediated degradation, pVHL protected TFAM against degradation by the mitochondrial protease Lon Peptidase 1 (LONP1). Consequently, cells with mutant pVHL, including variants associated with type 2C disease, showed decreased TFAM levels and reduced mitochondrial mass. Treatment with the protease inhibitor bortezomib rescued TFAM levels and sensitized the *VHL*-mutant 786-O RCC cell line to treatment with the kinase inhibitor sorafenib, both in vitro and in xenograft studies in mice [40]. Hence, TFAM levels correlate with sorafenib response, but the causality remains to be determined as both bortezomib and sorafenib are broad acting drugs. Interestingly, a role for *VHL* in mitochondrial biogenesis is in line with decreased mitochondrial mass observed in VHL disease-associated lesions, such as ccRCC, PHEO and paraganglioma, as well as in sporadic *VHL*-mutant RCC [41–44]. Further investigation should reveal if the decreased mitochondrial numbers in these lesions can be (partly) attributed to TFAM destabilization or to other consequences of pathogenic *VHL* variants, like increased HIF activity.

**Fig. 1:**
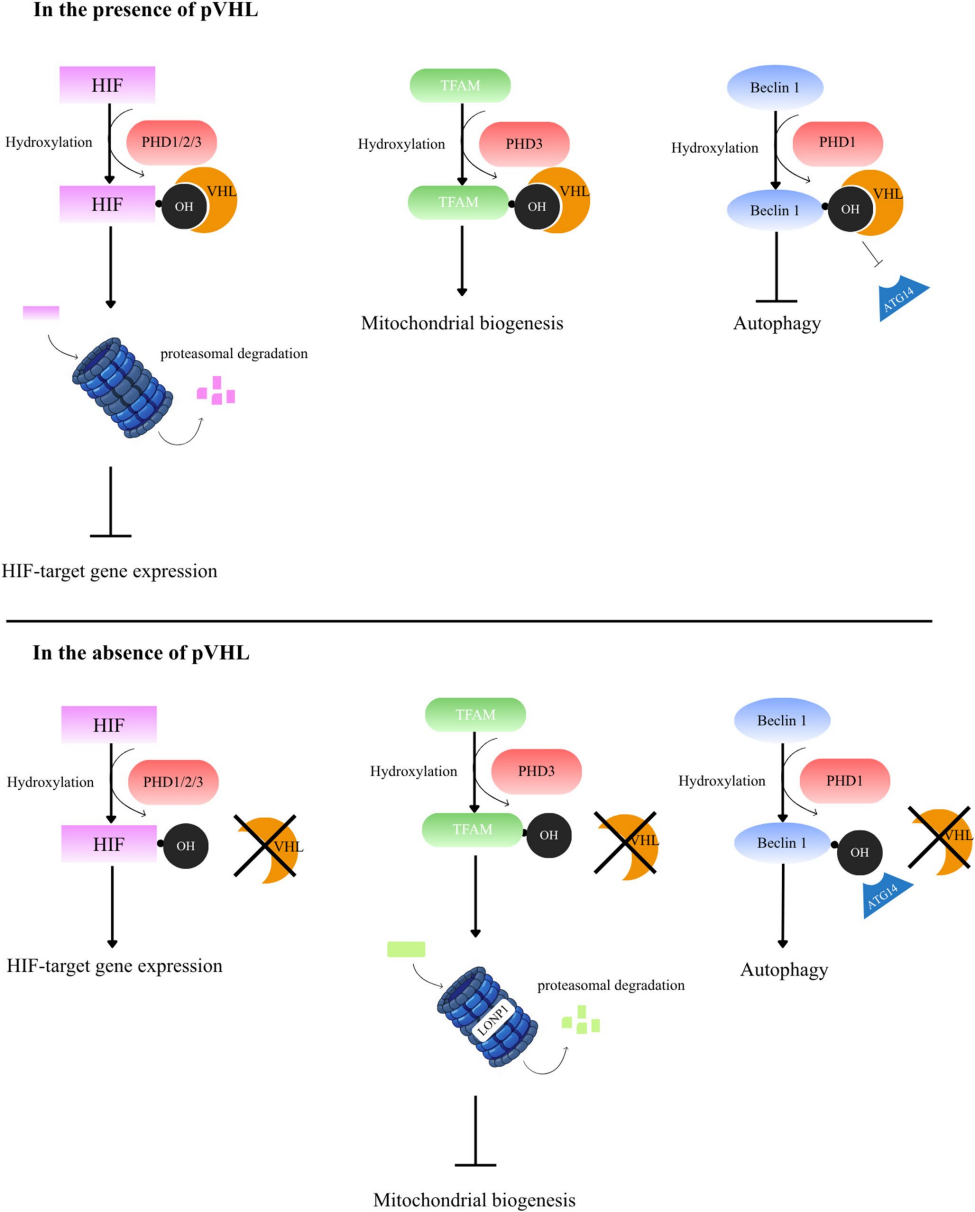
Schematic depiction of the regulation of HIF, mitochondrial biogenesis and autophagy by pVHL. Abbreviations: HIF, hypoxia-inducible factor; VHL, Von Hippel-Lindau; TFAM, mitochondrial transcription factor A; PHD, prolyl hydroxylase domain; LONP1, Lon Peptidase 1; ATG14L, Autophagy-Related 14-Like protein. Created with canva.com

Furthermore, pVHL was recently shown to function as a direct inhibitor of nutrient stress induced autophagy [45]. It disrupts formation of the autophagy activating complex containing Beclin1, Autophagy-Related 14-Like protein (ATG14L) and Vacuolar Protein Sorting 34 (VSP34), by direct interaction with hydroxylated Beclin1. Increased autophagy in *VHL* mutant cells could promote tumor formation by providing nutrients for tumor cell proliferation and metastasis [46]. Consistently, prevention of autophagy using the VSP34 inhibitor SAR405 reduced 786-O xenograft tumor growth in mice. A schematic depiction of the role of pVHL in the regulation of HIF, TFAM and Beclin1 can be found in Fig. 1.

Also, two recent genetic screens suggested a role for pVHL in repair of DNA double-strand breaks, which are dangerous DNA lesions with strong mutagenic potential if improperly dealt with [47, 48]. A DNA repair function of pVHL is in line with reports suggesting that pVHL promotes homologous recombination, a DNA double-strand break repair pathway [49]. The molecular mechanism by which pVHL would promote DNA repair is unclear, although one study indicated that the DNA repair defect in *VHL* mutant cells was dependent on HIF-1a [48]. Further investigation into this function of pVHL is warranted given the intricate connection between defective DNA repair and cancer development, as well as anti-cancer treatment response [50]. Finally, *VHL* is implicated to influence apoptosis, cell senescence, and metastasis, as extensively discussed in previous reviews [51–53]. The extent to which these functions are relevant in VHL disease has not been fully established.

Biallelic pathogenic variants of the *VHL* gene is generally regarded as a critical initiating event in the development of tumors. However, kidneys from individuals with VHL disease may harbor thousands of cells containing biallelic pathogenic *VHL* variants, yet only a fraction of these cells progress to form clinically relevant cysts or tumors [54]. This would suggest that ccRCC development requires additional variants on top of pathogenic VHL variants [55]. Other frequently mutated genes in VHL disease-associated ccRCC are *PBRM1, BAP1 * and * SETD2,* all of which are on chromosome arm 3p [56]. Whether inactivation of these genes contributes to ccRCC initiation or drives tumor growth at a later stage is not known. For other VHL disease-associated lesions there is currently limited data on the role of (secondary) variants in driving tumor development.

## Genotype–phenotype correlation

Attempts to document the risk profile of VHL disease based on genotype–phenotype correlations have been made since the late twentieth century, when a relationship between PHEO and germline missense pathogenic *VHL* variants was established [8, 57]. Given that a wide spectrum of VHL disease-associated lesions can arise from virtually any pathogenic germline *VHL* variant, it remains challenging to exclude specific genotypes from surveillance protocols. Therefore, it is important to note that the genotype–phenotype relationship in VHL disease can currently only be used for research purposes and not in clinical practise. Nonetheless, understanding genotype–phenotype correlations may facilitate a more personalized approach to clinical management in the future, enabling the development of patient-specific screening and care strategies [57–61]. For patients, this could also mean a better risk communication. However, subgroups sizes are often limited and direct comparison across studies remains challenging due to substantial heterogeneity in reported outcome parameters, such as the specific type of genetic alteration (e.g., single amino acid substitutions) [58], localization of the pathogenic variant within the VHL protein [59–61], and consequences on protein folding (e.g. truncating versus non-truncating) [62]. Chiorean et al. [63] developed a large-scale genotype–phenotype–driven machine learning model based on a systematic review, enabling standardized classification of *VHL* variants across studies and facilitating broader comparison. Despite this integrative approach, the results cannot yet be translated into clinically actionable strategies, particularly for tailoring individual surveillance based on genetic risk. Lastly, it should be noted that other factors (such as lifestyle factors) and variants in other genes outside the *VHL* gene may influence phenotypical expression of VHL disease and are not taken into accounts in the current genotype–phenotype studies.

A detectible germline pathogenic *VHL* variant is found in up to 95% of patients with a clinically determined diagnosis of VHL disease, as was described above. Hence, pathogenic variants in other genes (i.e. *ELOC* and *HIF2A (EPAS1))* can be responsible for a VHL disease-like genotype and genetic testing for these genes can be implicated in case of a suspicion for VHL disease without a pathogenic *VHL* variant [64, 65]. Firstly, rarely, pathogenic germline variants in the *ELOC* gene, encoding the ELOC protein which is part of the VCB complex, results in similar presentation to VHL disease [66, 67]. Somatic *ELOC* mutations in sporadic ccRCC are observed in around 0.6–3%, and result in slow growing tumors with a low risk for distance metastasis [68]. Secondly, pathogenic variants in the *HIF2A* (*EPAS1*) gene result in a characteristic triad of symptoms (PPGL, polycythemia and somatostatinoma), known as the Pacak-Zhuang syndrome [69]. Finally, mosaicism in VHL disease should be considered in the absence of a pathogenic *VHL* variant on regular screening, as patients with this condition have a similar phenotype [70]. Additional testing of VHL disease-related lesions or different germ layers can be considered in case of VHL disease-phenotype without detectable germline *VHL* variants [71].

## Screening and treatment of VHL disease-related lesions

Several national guidelines have been published with recommendations for screening of patients with VHL disease [72, 73]. Recommended screening interval including screening modality for different VHL disease-related lesions are summarised in Table 1 [72, 73]. An extended version of this table can be found in the recommendations from the VHL alliance [73]. Below, the most important considerations for screening and treatment for individual VHL disease related lesions are mentioned. Recent years also marked the discovery of belzutifan (see below). The approval of belzutifan for the treatment of VHL disease-associated CNS hemangioblastoma, ccRCC and pNET has created new therapeutic opportunities in VHL disease management. However, questions remain regarding the optimal timing of treatment initiation and duration. The recommendations below are based on current guidelines, but will likely evolve as more evidence becomes available about optimal treatment strategies with belzutifan.

**Table 1:** Recommended screening intervals per VHL related lesion

Tumor	Screening interval	Starting age	Screening modality
Retinal angioma	Annually	As early as possible	Fundoscopy
CNS hemangioblastoma	MRI every other year, yearly neurological examination	15 years, baseline scan at 10	MRI
Renal lesions	Every other year	15 years	MRI and/or CT
Pancreatic lesions	Every other year	15–65 years (in the absence of lesions)	MRI and/or CT
Pheochromocytoma	Annually	5 years	Serum/urine (nor)metanephrines measurement and MRI and/or CT
ELST	Every other year (as part of the CNS scan)	15 years	MRI and audiogram

Recommended screening interval for the most common VHL disease-associated lesions. Based on the Recommendations by the VHL alliance and Danish national guidelines [72, 73]. An extended version of this table is reported by the VHL alliance [73]. CNS, central nervous system; ELST, endolymphatic sac tumor

### Retinal angioma (RA)

RAs are the considered the first presentation in 43% of patients with VHL disease, reaching a cumulative probability of 80% by the age of 80 [74]. It is recommended to start annual screening as early as possible [72]. Caution in screening is required in early teenage years and twenties, for most new ocular manifestations develop during this period [72, 75]. The preferred treatment option for smaller (< 1.5 mm), mostly extrapapillary lesions is laser photocoagulation. For more complex (e.g. retinal detachment; epiretinal or vascular proliferation) or larger RA, surgery can be considered. Finally, juxtapapillary lesions, due to their proximity to the optic nerve head, are not suitable for ablation therapy. This can result in visual field loss and central scotoma. Given the fluctuating efficacy of other therapies such as photodynamic therapy with verteporfin, observational strategies must be considered as a suitable management for VHL disease-associated RA. Juxtapapillary lesions infrequently cause exudates that significantly impact the macula. In the case of RA with more severe exudation, photodynamic therapy, proton beam therapy, intravitreal VEGF antagonists or corticosteroids can be offered, but adverse effects (AE) may outweigh possible benefits [76].

### CNS hemangioblastoma

CNS hemangioblastomas occur in 60–80% of patients with VHL disease. It is recommended to start screening the CNS every other year from the age of 15 years old, with a baseline scan at 10 years old [72]. The development of symptoms depends mainly on the size and location of the lesions [77]. During surveillance visits, the neurological examination should be based on imaging and/or symptoms, rather than an extensive neurological examination [72]. With the unpredictable, stuttering growth pattern of most CNS hemangioblastomas it is recommended to only surgically treat fast growing tumors or after the development of early signs and symptoms, and not based solely on tumor size [78, 79].

### Renal lesions

RCCs are common in VHL disease, with up to 70% of patients developing at least one RCC before the age of 60 [80]. It is advised to start screening every other year from the age of 15 years [72]. The vast majority of these tumors (91%) can be characterised as of the clear-cell type [81]. Due to the potential for malignant transformation from VHL disease-related renal cysts, surveillance of cysts is important. Following detection of renal lesions, the characteristics, size and growth should be determined. To this end, the International VHL Surveillance Guidelines Consortium recommends increased MRI frequency (every 3–6 months) to determine the tumor kinetic growth for personalised follow-up scheduling [73, 82]. For small tumors (< 3 cm), active surveillance is recommended due to the low risk of metastasis. It is recommended to assess tumors > 3 cm for nephron-sparing surgical resection, which keeps the loss of nephrons to a minimum [82]. As an alternative for surgery in smaller lesions, local therapies like radiofrequency ablation can also be considered.

### Pancreatic manifestations

Pancreatic cysts or NETs are observed in around 60% of patients, with the prevalence of pNET ranging from 8% to 17% [83, 84]. Similar to CNS and kidney lesions, pancreatic lesions are advised to be screened every other year from the age of 15 years old [72]. The VHL Alliance recently (2022) published an updated recommendation for the treatment of pNETs in VHL disease [84]. Due to the rare occurrence of malignant formation from VHL disease-related cysts, the treatment of cysts should be based on symptomatology or in case malignancy cannot be ruled out.. Larger pNETs (> 2.8–3.0 cm) pose an increased risk for metastasis and it is therefore recommended to consider resection of pNETs 2–3 cm in diameter. In addition, due to the possible need for Whipple’s procedure if left untreated, it should be considered to resect tumors of the pancreatic head earlier.

### Pheochromocytoma

With an incidence of 10–20%, PHEO is a frequent manifestation of VHL disease [85]. PHEOs associated with VHL disease are categorised as cluster 1 (i.e. pseudohypoxic cluster) [86]. Annual measurements of plasma (nor)metanephrines are recommended from the age of 5 years old, due to their high sensitivity (up to 97%) and specificity (up to 96%) for the diagnosis of PHEO [87]. Cluster 1 PHEOs generally present with a noradrenergic phenotype (i.e. elevated normetanephrines and no/small increase in metanephrines). No consensus exists regarding the starting age of biochemical screening, but some advocate to start screening as early as 5 years of age [72, 88]. Plasma (nor)metanephrines measurements are preferred over 24 h urine collection in children due to increased burden. Imaging (CT, MRI or PET/CT) is only recommended in cases with elevated (nor)metanephrines. However, adrenals are also depicted with the regular screening for renal lesions [72, 89]. It is recommended to measure the blood pressure yearly in all patients, especially in those not screened for (nor)metanephrines. Treatment of local tumors includes cortical sparing or complete adrenalectomy, with preoperative treatment with alpha-adrenergic blockage to optimize hemodynamics and decrease postoperative cardiovascular complications [19, 72, 89–91]. Partial adrenalectomy should be considered for all VHL disease-associated pheochromocytoma in an attempt to prevent adrenal insufficiency [92].

### Endolymphatic sac tumors (ELST)

ELSTs are tumors originating from the epithelial cells of the endolymphatic sac located within the vestibular aqueduct [93]. ELST can be evaluated together with the CNS lesions (every other year, from the age of 15 years) [72]. Though not amongst the most common of manifestations with an incidence between 4% and 16%, ELST pose an increased risk of hearing loss when left untreated [94]. Hence, early diagnosis and treatment is critical for maintaining proper hearing. Since auditory loss can indicate tumor formation and be caused by small lesions invisible on routine imaging, yearly audiograms are recommended. There is no consensus in guidelines as to the start and frequency of audiograms. Preferred treatment for ELST is surgery, even for small lesions, as irreversible hearing loss can be a serious consequence of these tumors [72, 94].

### Epidydimal cystadenoma (EC)

ECs are relatively common, benign lesions presenting in around 54% of male patients. Due to their benign course and absence of symptoms, no routine screening is advised. Patients are advised to self-examinate their scrotum for enlargements suspicious of testicular cancer, similar to the advice in the general population. Ultrasonography can be used to determine if these enlargements are a result of ECs [73]. Due to the benign course of most ECs, no surgical resection is recommended. However, they should be followed for growth and patients are to be instructed to seek medical help in case of scrotal pain. Though unknown for VHL disease-associated ECs, sporadic cases have not been associated with a decrease in fertility [95–97].

### VHL disease and pregnancy

There is only scarce data describing the effects of pregnancy on the development of VHL disease-related lesions [98–100]. These studies included small sample sizes and often did not correct for maternal age. An increased growth rate of VHL associated hemangioblastomas has been reported in one cohort [100].

### VHL disease in paediatric patients

The probability of developing VHL disease-associated lesions substantially increases with age [1]. Hence, delayed screening increases the chance that lesions remain undiagnosed. Although VHL manifestations can occur at young age, the clinical benefits of screening should be weighed against burden of repeated diagnostics in this population. For example, CNS HB have been found in 9 year old children, but these did not require treatment with a likelihood of 95% in children under 13 years in a German cohort with 99 children[101]. As, children often require sedation at young age to undergo an MRI, surveillance is recommended to start at 11 years of age.

## Future diagnostics, screening and prognostics

### Blood/urinary biomarkers

Currently, blood or urinary biomarkers only play a role in the detection of PHEOs, yet not for the detection of any other VHL disease-related lesions [72].

Several metabolites have been studied in the context of VHL disease. Cellular metabolic changes in cancer cells result in alteration of the normal systemic metabolite concentration, making metabolites a potential prognostic markers in cancer [102]. Downregulation of N2,N2-dimethylguanosine was a significant predictor for the onset of ccRCC in VHL disease (p < 0.001) [103]. Hypoxanthine, dodecanoylcarnitine, and 4-Dihydroxy-2-hydroxymethyl-1-pyrrolidinepropanamide (4D2h1p) show relationships with the early occurrence of CNS hemangioblastoma [103]. Trehalose was significant for the prediction of earlier onset of pancreatic cysts of tumors [103]. Only few biomarker studies have been performed in patients with VHL disease. Urinary miR-542-5p has been reported to be of interest for the detection of VHL disease-related ccRCC as concentrations declined significantly after ccRCC removal, suggesting an association with ccRCC tumor mass, yet further validation is needed [104].

### Imaging

Besides screening, MRI imaging could potentially be used for VHL disease-related tumor risk stratification. Regular MRI scans are recommended for all patients, but currently only size and growth rate are used to select patients for treatment. Other predictors are being studied but are not used in clinical practice yet. For example, apparent diffusion coefficient (ADC) on MRI is positively correlated with tumor doubling time and inversely correlated with growth [105]. Other radiomics that are promising for the prediction of volume doubling time (VDT) are the region of interest (ROI) longest axis, grey-level cooccurrence matrix (GLCM) and grey level size zone matrix (GLSZM) [106].

Although MRI is currently the golden standard for the visualisation of VHL disease-associated lesions several PET-tracers have been studied in the context of VHL disease [107]. Especially, ^68^Gallium[68 Ga]-DOTA peptide PET/CT was found to be superior in the visualisation of lesions in two studies [108, 109]. In general, PET/CT offers advantages as it results in lower radiation exposure and is non-nephrotoxic, contrary to conventional contrast used on CT [107]. This is particularly beneficial for VHL patients, who undergo frequent scans and are at risk of kidney failure as a result of repeated surgical renal interventions [110, 111].

## Systemic therapy

### HIF-2α targeted therapy (belzutifan)

Until 2021, pazopanib, a tyrosine kinase inhibitor (TKI) primarily inhibiting angiogenesis, was the only systemic therapy specifically studied for the treatment of VHL disease-associated tumors [4, 112]. Pazopanib has primarily been used for the treatment of sporadic RCC and has hence also been studied as such [113, 114]. The efficacy of pazopanib for the treatment of VHL disease was studied in 2018 by E. Jonasch et. al. in a non-randomised control trial with 31 patients [115]. A response was observed in 52% of RCC, 53% of pancreatic lesions and 4% of HBs. Most common side effects were diarrhoea, fatigue and elevated liver enzymes, though often mild (grade 1–2).

A major breakthrough, however, has been the development of belzutifan, a small-molecule, second generation HIF-2α inhibitor. Belzutifan binds to HIF-2α, which prevents its binding with HIF-1β. Ultimately, this decreases the transcription of downstream molecules responsible for tumor growth and formation, such as VEGF and cyclin D [36, 116].

Reproductive studies in rats have shown that administration of belzutifan during organogenesis leads to high fetal toxicity in all dosages [120]. Hence, it is important to verify pregnancy status prior to initiation with belzutifan and inform patients on fetal risk associated with this drug. Belzutifan decreases the effectiveness of hormonal contraception and therefore the need for adequate non-hormonal contraception should be discussed with patients (and their partners) of fertile age during treatment with belzutifan.

AEs reported were primarily grade 1–3 (Table 2). Most frequently noted AE the use of belzutifan include anemia (89–100%), fatigue (64–100%), dizziness (25–100%) and headache (25%). Anemia can most likely be attributed to the decreased transcription of EPO following HIF-2α inhibition and had to be treated with EPO stimulating agents (12 patients, 20%) or blood transfusions (4 patients, 7%) in the LITESPARK-004 trial [121]. AEs led to the cessation of treatment in 26 patients (43%), and a dose reduction in 9 patients (15%).

The first published clinical experiences with the use of belzutifan for the treatment of CNS lesions seem to confirm the results from Jonasch et. al. [122, 123].

Belzutifan was approved by the U.S Food and Drug Administration (FDA) in 2021 and the European Medicines Agency (EMA) in 2025 for the treatment of VHL associated ccRCC, CNS hemangioblastomas and pNET for which localised procedures are not feasible [124, 125]. It has also been approved for the treatment of sporadic (metastatic) ccRCC after treatment with a programmed death-ligand 1 (PD-L1) inhibitor and a VEGF-TKI, like sorafenib [126]. For both indications, the recommended dosage is 120 mg daily administered orally [120].

A future challenge is the potential development of acquired resistance to belzutifan [127]. Since its clinical introduction in 2021, resistance patterns remain largely unknown and are under investigation. Suggested mechanisms include direct reduced affinity from HIF-α to belzutifan through *G323E* pathogenic variants or indirect through higher PHD affinity to HIF-α following *F446L* pathogenic variants. The importance of dual therapy is further underscored by the observation that, in mouse models, maintenance of VEGF and cyclin D1 expression resulted in almost a complete resistance to belzutifan. To explore this concept further, the LITESPARK-011 aims to determine the efficacy of belzutifan combined with tyrosine kinase inhibitor Lenvatinib and cabozantinib [128]. Previous reviews have summarised the other current ongoing trials regarding combination therapy with belzutifan. Amongst them are the LITESPARK-022 (double-blind, randomized phase 3 clinical trial on belzutifan and pembrolizumab vs. placebo and pembrolizumab), LITESPARK-003 (phase 3, randomized trial on belzutifan and zanzalintinib vs. cabozantinib), NCT04736706 (phase 3 clinical trial on belzutifan and lenvatinib and pembrolizumab vs. quavonlimab and pembrolizumab and Lenvatinib vs. pembrolizumab and Lenvatinib) [129, 130]. Currently, these combination studies are primarily focussed on the treatment of ccRCC. 

Belzutifan was first clinically investigated for VHL disease by Jonasch et.al in a single arm, phase 2 clinical trial (LITESPARK-004). The most important findings of the LITESPARK-004 study including follow-up data of the same patient population are summarized in Table 2 [36, 117–119]. In ccRCC, the primary end point of the study, an objective response of 49% was observed, with another 49% having a best response of stable disease, and 3% experiencing disease progression. For CNS hemangioblastomas specifically, the decision to treat with belzutifan should not be based on the presence/absence of cystic components, as this does not influence treatment outcome [119]. Consistent with the results of this trial, the authors of the articles recommend considering treatment with belzutifan in all VHL disease-related lesions. However, the limited sample size and absence of a randomized clinical controlled trial comparing belzutifan to currently available treatment options should be taken into account when starting belzutifan.

**Table 2:** Summary of the most important findings of the LITESPARK-004 study

	Jonasch et al. [36]	Else et al. [117]	Iliopoulos et al. [119]		Wiley et al. [118]	
Tumor type	ccRCC	pNETs	CNS HB solid and cystic	CNS hemangioblastoma solid component only	RA	
No. of patients	61	22	50	25	12 (16 eyes with active RA)	
Median follow-up time (months)	21.8	37.8	38.0	38.0	37.3	
*Beste response n* (%)					*Response n* (%)	
Complete response	0	7 (32)	4 (8)	1 (4)	Improved	12 (100%)
Partial response	30 (49)	13 (59)	18 (36)	18 (72)	Stable	0
Stable disease	30 (49)	2 (9)	23 (46)	5 (20)	Progressed	0
Disease progression	0	0	3 (6)	0	Not evaluable	0
Not evaluable	1 (2)	0	2 (4)	1 (4)	No assessment	00
*AE n* (%)						
Any grade treatment related AE	61 (100)	61 (100)	50 (100)	NA	12 (100)	
Grade 3–5	20 (33)	27 (44)	23 (46)	NA	4 (33)	

Summary of the most important findings of the LITESPARK-004 study, including best response to treatment with belzutifan and AE. In the follow-up study from the LITESPARK-004 focussing on CNS lesions, a distinction was made between CNS cysts and hemangioblastomas. The author made a division between patients with hemangioblastomas (measurable (longest diameter > 1 cm) and non-measurable (longest diameter < 1 cm), including possible cystic components) and patients with only measurable solid lesions. ccRCC, clear cell renal cell carcinoma; CNS, central nervous system; pNET, pancreatic neuroendocrine tumor; RA, retinal angioma; AE, adverse event

### Vorinostat

Another treatment option currently under investigation for the treatment of CNS hemangioblastomas in VHL disease is the administration of histone deacetylase inhibitors (HDACIs), specifically vorinostat. This drug influences a large number of cellular processes and proteins [131], yet the effect on the chaperone heat shock protein 90 (Hsp90) is of particular importance in regards to VHL disease.

Two chaperone heat shock proteins are important in the metabolism of pVHL: Hsp70 is involved in proper protein folding and, to a lesser extent, pVHL breakdown. Contrary, Hsp90 targets pVHL for degradation. Wild-type pVHL has a strong affinity for Hsp70, whereas missense mutant pVHL binds stronger to Hsp90 leading to faster degradation of missense mutant pVHL as compared to wild type pVHL. Vorinostat binds Hsp90 and subsequently is able to increased mutant pVHL half-life from 2.5 to 10.3 h [132]. This effect of vorinostat appear to be missense variant specific.

Analysis of resected hemangioblastoma in patients administered 400 mg vorinostat 7 days before removal showed increase pVHL expression, with decreased HIF-2α and associated downstream proteins (i.e. VEGF-A and GLUT-1). Reduced tumor growth after administration of vorinostat has been reported in murine models. However, no data is available yet on effects of vorinostat on tumor growth in humans [133].

## Conclusion

Recent years have been marked by major successes regarding the treatment of VHL disease demonstrated by the registration of belzutifan in 2021. Over 3 years of follow-up now indicate promising results for the treatment of VHL disease. However, not all patients respond to belzutifan, possibly due to the HIF-independent effects of pVHL. The discovery of these roles of pVHL could open up new potential pathways that could be targeted for future treatment options. As of now, guidelines recommend patients with VHL disease to undergo screening using mainly MRI or CT, as current genotype–phenotype classifications do not allow for personalized screening or prognostication. To this end, there is a need for biomarkers and specific radiomics to potentially be used for personalised screening and treatment. Continued research into the molecular causes of VHL disease, combined with advances in imaging and personalized surveillance, will be essential to improving outcomes and personalised treatment.

## Data Availability

All data resulting from literature search is available on Pubmed.
